# Self-rated oral health among elderly patients attending a university dental hospital in Thailand: a telephone-based cross-sectional survey study

**DOI:** 10.7717/peerj.14191

**Published:** 2022-10-10

**Authors:** Nithimar Sermsuti-anuwat, Narongrit Nampikul, Rawitsara Suwannimit, Weerachon Panthueng

**Affiliations:** 1 Academic Affairs Division of the Faculty of Dentistry, Chulalongkorn University, Bangkok, Wangmai, Pathumwan, Thailand; 2 Bachelor Programme in Doctor of Dental Surgery of the Faculty of Dentistry, Chulalongkorn University, Bangkok, Wangmai, Pathumwan, Thailand

**Keywords:** Geriatric dentistry, Oral health, Oral health promotion, Self-rated oral health, Subjective oral functions

## Abstract

**Background:**

Oral health perception is an influential predictor of both current and future health among the elderly. However, limited research has focused on self-rated oral health among older patients attending tertiary dental care. Therefore, this study aimed to explore the potential factors associated with self-rated oral health among elderly patients attending a university dental hospital in Thailand.

**Methods:**

This telephone-based cross-sectional study was carried out among elderly patients older than 60 years who attended at least one dental visit at the university dental hospital in 2020. Hospital numbers (HN) were used to identify eligible candidates for this study. We calculated the sample size by assuming a finite population of 70,028 elderly patients with valid telephone numbers. The minimum sample required for this study was 398 participants. Trained interviewers conducted telephone calls between July 2021 and January 2022 using the validated modified oral health questionnaire. Self-rated oral health was assessed using a conventionally used global oral health question: “How would you describe your dental health?” with three response options: good, fair, and poor. Descriptive statistics, Fisher’s exact test, and binary logistic regression were performed to analyze the data.

**Results:**

A total of 836 telephone numbers were called. There were 402 (48.10%) elderly patients who agreed to and completed the telephone interview. Most of the study participants were women (61.4%) between 61–74 years of age (83.1%) with a mean age of 69.18 years. Bivariate analyses showed associations between poor self-rated oral health and lower subjective oral functions: chewing discomfort (*p* < 0.001) and speaking discomfort (*p* = 0.013). However, the multivariate regression model indicated a significant association between poor self-rated oral health and chewing discomfort (*p* < 0.001). Therefore, elderly patients with chewing discomfort were more likely to perceive poor oral health.

**Conclusions:**

These findings indicate that difficulty chewing could be a potential factor influencing self-rated adverse oral health among older patients attending the university dental hospital. Furthermore, our study adds that the predictive power of a single-item self-measurement supports its value as a standard measure to predict oral health risk in tertiary care institutions, as well as primary care settings and community-based survey research. Therefore, healthcare providers should routinely evaluate self-rated oral health among elderly patients to detect early signs and symptoms of oral health problems, assess the success of dental treatments, and monitor general health and well-being.

## Introduction

The world population of adults aged 60 or older is estimated to double by 2050 and triple by 2100 compared to 2017. As a result, the number of older people worldwide is expected to rise from 962 million in 2017 to 2.1 billion in 2050 and 3.1 billion in 2100. Thailand is considered one of the most rapidly aging societies worldwide as the the Thai population over 60 years of age is expected to double between 2015 and 2050 (The United Nations, 2017).

According to the 8th National Survey on Oral Health in Thailand (8th NSOHT) report, Thais are at high risk for health problems, chronic diseases, and non-communicable diseases, including poor oral health (MPH et al., 2018). In addition, older Thai adults, aged 60 to 70 years, have just 18.6 remaining teeth and this number decreases to 10 remaining teeth among adults aged 80 to 85 years (MPH et al., 2018). These findings show that elderly Thais are more likely to have fewer teeth remaining, particularly in the older age range.

Recent studies indicated that, due to age-related decline, physical restrictions, and limited oral health literacy, older Thai adults are less able to control dental plaques and are more likely to have inadequate oral hygiene, poor oral health, and eventually fewer remaining teeth in older ages (Piyakhunakorn & Sermsuti-Anuwat, 2021a; Piyakhunakorn & Sermsuti-Anuwat, 2021b; Sermsuti-Anuwat & Piyakhunakorn, 2021).

Although the majority of healthy, independently healthy older Thai adults regularly used fluoride dentifrice to brush their teeth daily, many negative oral health behaviors were identified, such as smoking, drinking alcohol, and irregular dental visits (MPH et al, 2018 ). Furthermore, the 8th NSOHT also mentioned that only 24.9% of elderly Thais living in the community considered themselves as having good oral health (MPH et al., 2018). Similar findings in other countries have reported that older people with negative oral health behaviors are more likely to perceive themselves as having poor oral health (Moon, Heo & Jung, 2020; Ugarte et al., 2007).

Global self-rating of oral health is a single-item measure with 3–5 self-rated options from excellent to very poor. Self-rated oral health helps summarize how people evaluate their own oral health (Locker, Maggirias & Wexler, 2009; Locker, Wexler & Jokovic, 2007). Older adults are more likely to be confused by complicated and lengthy questions, including discomfort with a complete oral clinical examination; therefore, self-rated oral health can be used as a quick, but effective tool for assessing oral health among the elderly (Hakeem, Bernabé & Sabbah, 2021). Current studies have supported using these single-item measures instead of lengthy indexes in low-resource settings (Lawal, 2015), where there are limited healthcare providers, or when a complete oral examination is infeasible (Hakeem, Bernabé & Sabbah, 2021; Lundbeck, Smith & Thomson, 2020).

In addition, numerous studies have observed associations between self-rated oral health and multiple variables. Moon, Heo & Jung (2020) found that self-rated oral health was related to demographic characteristics, socioeconomic factors, oral health-related behaviors, and oral health-related quality of life. Ugarte et al. (2007) found relationships between educational level and self-perception of oral health, as education may lead to appropriate oral health behaviors among the elderly. Self-reported oral health is also associated with clinical oral health. A study by Lundbeck, Smith & Thomson (2020) in New Zealand found that the self-reported oral health of older adults in aged residential care aligned accurately with clinical indicators of oral health.

In terms of subjective oral functions, many concurrent studies have found statistically significant associations between self-perception of oral functions and self-rated oral health. For example, a study in Korea found that chewing and speaking abilities were associated with self-rating of oral health in older Koreans (Moon, Heo & Jung, 2020). Arantes & Frazão (2018) found that difficulty speaking was related to negative self-perception of oral health among indigenous people in central-west Brazil. Furthermore, many existing publications have reported a significant relationship between self-rated oral health and subjective chewing ability (Martins et al., 2011; Ugarte Cabo et al., 2006; Ugarte et al., 2007; Wright et al., 2019). Several recent studies explained that poor mastication probably worsens general health outcomes among the elderly (Kiesswetter et al., 2019; Lindroos et al., 2019; Okura, Ogita & Arai, 2020; Ramsay et al., 2018; Saintrain et al., 2018).

Moreover, self-rated oral health is a comprehensive measure that represents an individual’s experience with their oral health condition that might differ among patients of different ethnicity (Arcury et al., 2013), social capital (Aida et al., 2011), or cultural values. Martins et al. (2011) explained that resilient older participants might be more likely to self-rate good oral health despite having some legitimate oral health problems.

Conflicting findings have been reported on the relationship between self-rated oral health and demographic characteristics for example, a Korean study by Moon, Heo & Jung (2020) reported that age and sex were associated with self-perception of oral health. On the contrary, many studies in different countries did not report an association between self-rated oral health and age or sex (Ekanayke & Perera, 2005; Ugarte Cabo et al., 2006; Ugarte et al., 2007; Dahl, Calogiuri & Jönsson, 2018). Additionally, regarding dental attendance among older people. Studies by Ugarte Cabo et al. (2006) and Ugarte et al. (2007) found that attending dental visits was correlated with self-reporting good oral health. In contrast, a study among older Norwegians by Dahl, Calogiuri & Jönsson (2018) and a study in Sri Lanka by Ekanayke & Perera (2005) found no association between self-reporting good oral health and having a dental appointment in the last twelve months.

An explanation of different study results has explained by Locker, Wexler & Jokovic (2007) and Locker, Maggirias & Wexler (2009), indicate that various frames of reference may provide different meanings of self-rated oral health, so the definition of good self-rated oral health may also differ among respondents. Self-rated oral health likely differs based on the respondent’s emotional state, physical state, demographic characteristics, economic status, underlying diseases, or health-related behaviors; those who report having poor oral health probably use a somewhat different frames of reference in their self-evaluation than those who report positive oral health, inducing inconsistent findings in research publications (Locker, Maggirias & Wexler, 2009; Locker, Wexler & Jokovic, 2007). Despite these inconsistencies, previous studies have suggested that self-rated rating is commonly used to predict both current and future health factors among the elderly (Balasubramanian et al., 2021; Hakeem, Bernabé & Sabbah, 2021; Lundbeck, Smith & Thomson, 2020). Therefore, it is important to consider the different frames of reference among study participants.

Current studies on self-rated oral health shows varied results. Most existing studies were conducted among older people from the community or in residential care (Dahl, Calogiuri & Jönsson, 2018; Ekanayke & Perera, 2005; Hakeem, Bernabé & Sabbah, 2021; Lundbeck, Smith & Thomson, 2020; Martins et al., 2011; Moon, Heo & Jung, 2020; Ugarte Cabo et al., 2006; Ugarte et al., 2007; Wright et al., 2019). It is possible that these elderly participants rarely sought oral treatments and thus approached the questions with a different frame of reference than the older adults who regularly attended dental visits.

Very few studies used a single-item rating as an oral health assessment tool among older patients (Balasubramanian et al., 2021; Saintrain et al., 2018). In addition, there is a lack of research focused on self-rated oral health among elderly Thais in a university dental hospital. We found only one descriptive study observed among patients registered at a university dental hospital in southern Thailand in 1992 (Benjakul & Chuenarrom, 2000).

This study aimed to explore the factors potentially associated with self-rated oral health among elderly patients who attended at least one dental visit in the last twelve months (in 2020) at the dental hospital of the Faculty of Dentistry of Chulalongkorn University (the university dental hospital). The results of our study provided necessary information to improve routine dental services and also highlight the importance of monitoring risk factors for poor oral health conditions among elderly patients in our university dental hospital and in other healthcare settings throughout Thailand. Additionally, these findings could help promote oral health among the older population in similar contexts in other countries.

## Materials & Methods

A telephone-based cross-sectional survey study was conducted between July 2021 and January 2022. We used the telephone interview method because it is considered a valuable and low-cost method to obtain a wide range of data when recruiting participants from a broad area (Copp et al., 1998). The Ethics Review Committee of the Faculty of Dentistry, Chulalongkorn University, approved the study protocol and the consent form (Ethical Application Ref: HREC-DCU 2021-039). Participants received a brief overview of the study investigation and information regarding confidentiality from trained reviewers. Participants were advised that they could withdraw from the study at any time. All interview materials were anonymized so that respondents could not be identified, and efforts were made to ensure that no additional interview information was published anywhere. All participants completed a verbal informed consent prior to the telephone interview. The study reporting was based on the STROBE statement: Strengthening the Reporting of Observational Studies in Epidemiology (Von Elm et al., 2014).

### Study population

All patients at the university dental hospital must be registered in the dental record system with general patient information and valid phone numbers before receiving dental care services. Thus, all phone numbers and patient information for this telephone survey were acquired from the dental record system at the university dental hospital. We then used the hospital number (HN) of elderly patients older than 60 years who registered for and attended a dental visit in 2020 to identify eligible candidates for this study. There were 70,028 patients who met the study criteria with valid telephone numbers, and these telephone number lists were established as a sampling frame of the target population.

### Sample size calculation

We calculated the minimum sample size needed by assuming a two-sided test with a significance level of 0.05 and a power of 80%, using the Yamane formula: *n* = *N*/1 + *Ne*^2^ (Yamane, 1967) and a finite population of 70,028 patients to calculate the required sample size. When n = sample size, *N* = 70,028 (Population size from total of patients with aged over 60 years, who had dental treatments in the Faculty of Dentistry Chulalongkorn University in 2020), e = Margin of error of 0.05. This means the sample size formula for our study was: *n* = 70,028/1+ (70,028 × 0.05^2^) = 397.7. The minimum sample size necessary for this study was 398 participants.

Proportionate stratified random sampling was used to ensure that the patient samples from each month, represented the whole sample population of the research study. Of the 70,028 valid telephone numbers, 12 groups were classified according to the months when patients attended dental visits. Telephone numbers (approximately 7,000 numbers/month) were labeled in ascending order. We used an online “Random Number Generator” by Furey (2006) to randomly choose phone numbers and generate 100 numbers without repetition for each group. Then, the randomized phone numbers were called only once in order to get the fastest response from the most prticipants. Patients who agreed to participate and had completed all interview questionnaires were included in the study. Phone calls were made until the study reached its target sample size.

### Eligibility criteria for participants

This study recruited men and women over 60 years of age if they were registered in the dental record system and had attended at least one dental visit at the university dental hospital between 1 January 2020 and 31 December 2020. Exclusion criteria were (1) individuals with disabilities; (2) individuals with hospital recorded history of severe chronic diseases such as uncontrolled hypertension (blood pressure >160/100 mmHg), uncontrolled diabetes (blood glucose levels >180 ml/dL), liver disease (bleeding problems), kidney disease (bleeding tendency), or blood diseases: hemophilia and congenital bleeding disorders; (3) an inability to communicate in Thai, or (4) individuals who were unwilling to participate.

### Variables

The independent variables were the following:

(1) Sociodemographic characteristics: age, sex, educational level, monthly income, adequate dental insurance
(2) Oral health-related behaviors: frequency of toothbrushing, duration of toothbrushing, compliance with dental appointments, smoking, and drinking alcohol
(3) Subjective oral functions: chewing ability and speaking ability

The dependent variable was self-rated oral health.

### Measurements

We used a validated modified oral health (MOH) questionnaire as a telephone interview questionnaire. The MOH questionnaire was adapted from the standard oral health questionnaire for older adults published in the 8th NSOHT report (MPH et al., 2018). In the MOH questionnaire, most variables were grouped into two possible answers (*i.e.*, age, 61–74 years or ≥ 75 years) in order to address lengthy questions and participant confusion. The authors received permission to use this instrument from the director of the Office of Dental Public Health, Bureau of Dental Health, Department of Health, Ministry of Public Health, Thailand.

The MOH questionnaire is made up of three sections. Section 1 consisted of four questions to collect sociodemographic data: respondents self-reported their age (61–74 years/ ≥ 75 years), sex (female/male), educational level (>primary education/ ≤ primary education, and monthly income (≥ 15,000 Thai Baht [437 US dollars]/<15,000 Thai Baht).

Section 2 contained five questions to collect oral health-related behavior data: respondents self-assessed the frequency of toothbrushing (≥ twice daily/<twice daily), duration of toothbrushing (≥ 2 min/<2 min), compliance with dental appointments (yes/no), smoking habit (never/sometimes/daily), and alcohol consumption (never/occasionally/daily).

Section 3 consisted of three questions: two questions to assess subjective oral functions and one to assess oral health perception. The questions to evaluate subjective oral functions were: “How would you rate your chewing ability?” (comfortable/fair/uncomfortable) and “How would you rate your speaking ability?” (comfortable/fair/uncomfortable). For self-rated oral health, we used the conventionally global self-rating of oral health question (Locker, Wexler & Jokovic, 2007): “How would you describe your dental health?” (good/fair/poor).

### Quality of the questionnaire

The validity and reliability of the MOH questionnaire were tested. The index of item-objective congruence (IOC) was analyzed for content validity testing. The IOC was acceptable with a value of 0.77. A pilot test was conducted in order to test the reliability of the questionnaire. A total of 30 people over 60 years of age, who were not patients of the university dental hospital participated in the pilot test in order to avoid overlap with eligible study participants. The Cronbach’s alpha coefficient was analyzed to test internal consistency reliability. The Cronbach’s alpha value of the MOH questionnaire was also acceptable at 0.70.

### Training interviewers

The three interviewers (NN, RS, and WP) were trained by the he principal investigator (NS) with a 3-hour training program before conducting telephone interviews. The training program content consisted of basic interview techniques including guidance to read the questions as written with a courteous and gentle tone of voice and an appropriate explanation. In order to reduce interviewer bias, the trained interviewers had never previously contacted the participants before conducting phone interviews.

### Data analysis

All quantitative data were analyzed using the SPSS software (version 28; SPSS, Inc., IBM, Chicago, IL, USA). This study descriptively analyzed the following variables: sociodemographic characteristics, behaviors related to oral health, subjective oral functions, and self-rated oral health.

Subjective oral functions (chewing ability and speaking ability) were classified into two groups: “comfortable” as “comfort” and “fair/uncomfortable” as “discomfort.” In our study, the participants were older patients attending in a tertiary care setting. Most of the patients had regular dental visits and were expected to be able to improve their oral health condition with follow up dental visits. Therefore, we intended to discriminate “comfortable” from “fair/uncomfortable” to determine the quality of dental care services in our dental hospital. Ideally, all patients who had regular dental visits, should self-report with “comfortable” chewing and speaking abilities. However, if they self-reported “fair” or “uncomfortable” chewing and speaking abilities, this might reflect problems related to oral functions that need to be urgently addressed.

Self-rated oral health was dichotomous with two levels: “good/fair” as “good oral health (0)” and “poor” as “poor oral health (1).” We used the classification of self-rated oral health in the same way as several previous publications (Dahl, Calogiuri & Jönsson, 2018; Ekanayke & Perera, 2005; Martins et al., 2011; Ugarte Cabo et al., 2006; Ugarte et al., 2007).

Fisher’s exact tests identified associations between self-rated oral health and categorically independent variables; variables with a *p* < 0.25 were included in binary logistic regression analyses using the enter method. Simple bivariate logistic regression analyses were performed to identify the independent variables that with a *p* < 0.25. We then included these related variables in the multivariate logistic regression analysis. In the regression models, the crude and adjusted odds ratios (OR) with a 95% confidence interval (CI) were calculated. A *p* < 0.05 was considered statistically significant. The Hosmer-Lemeshow test was used to ensure the goodness of fit of the final model; a non-significant Chi-square is desirable (Pett, 2016). Multicollinearity was checked using the variance inflation factor (VIF), a tolerance value below 0.1, and a VIF value above 10 indicating multicollinearity problem (Miles, 2014).

## Results

**Figure 1:**
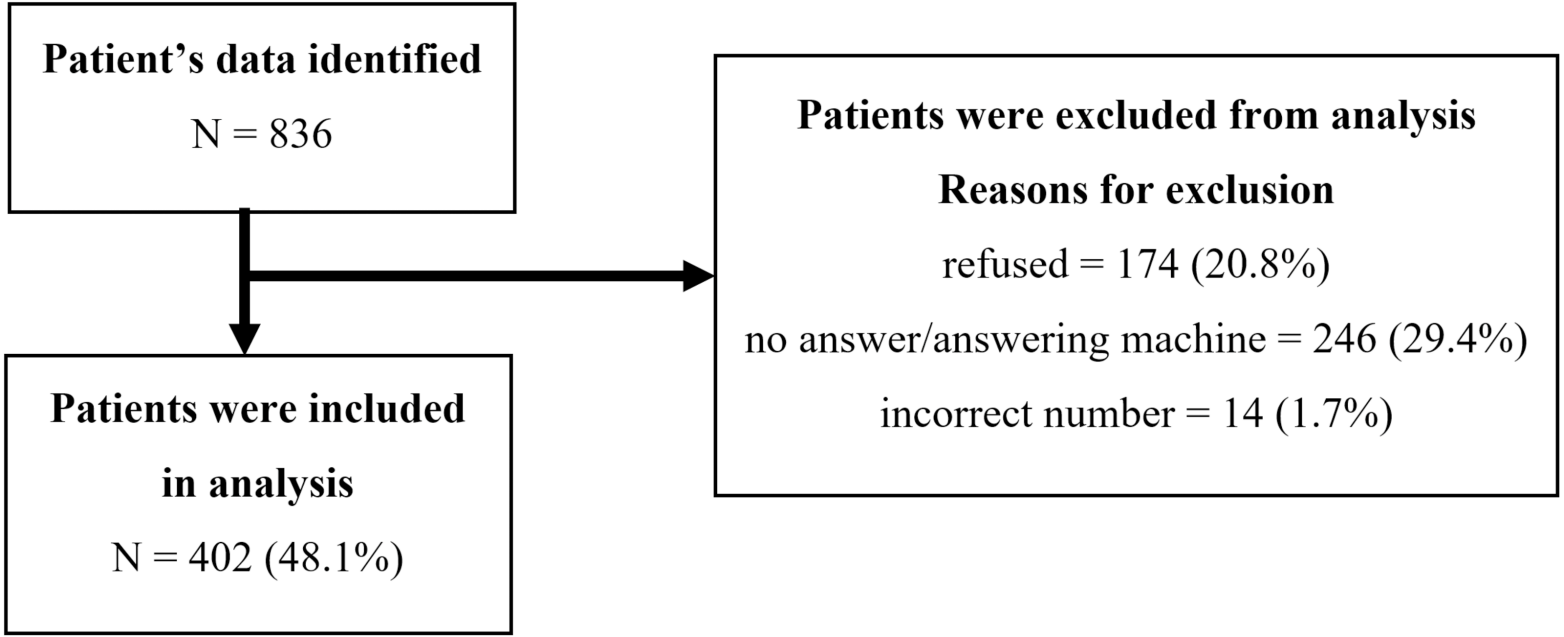
A flow diagram of study participants.

We called a total of 836 older adults for our telephone survey: 402 (48.1%) agreed to participate and completed telephone interviews; 174 (20.8%) refused; 246 (29.4%) did not answer, continued to ring, or went to viocemail; and 14 (1.7%) were incorrect phone numbers (Fig. 1). Our response rate (48.1%) is similar to an earlier telephone survey study among elderly Thai patients using resource data from the same university dental hospital, with 52.4% responding (Hongpaitoon, Siriwatana & Sermsuti-Anuwat, 2022).

Table 1 presents the distribution of related variables of 402 participants ranging from 61 to 94 years old, with a mean age of 69.18 years. For sociodemographic characteristics, the majority of our respondents were women (61.4%), between 61–74 years of age (83.1%), with higher than primary education (84.1%), and a monthly income of at least 15,000 Thai Baht (437 US dollars; 57.2%). Regarding oral health behaviors, the majority of participants with at least one tooth (*N* = 397) reported toothbrushing frequency twice a day (94.5%). Furthermore, most of the participants reported a toothbrushing duration of at least 2 min (80.6%) and a compliance with dental appointments (74.4%). In addition, the participants said that they had never had a habit of smoking or drinking alcohol (>90.0%). Concerning subjective oral functions, 59.5% reported feeling comfortable chewing, and 90.3% felt comfortable speaking. However, only 32.1% of the participants self-reported good oral health.

Fisher’s exact tests indicated statistically significant associations between self-rated oral health and subjective oral functions: chewing ability (*p* < 0.001) and speaking ability (*p* = 0.019; Table 2). On the contrary, we did not observe a statistically significant association between self-rated oral health and age, sex, educational level, monthly income, toothbrushing frequency, duration of toothbrushing, compliance with dental appointments, smoking, and or alcohol consumption. However, the following related variables with a *p* < 0.25 were included in the regression analyses: sex (*p* = 0.168), educational level (*p* = 0.226), monthly income (*p* = 0.131), toothbrushing frequency (*p* = 0.176), compliance with dental appointments (*p* = 0.175), drinking alcohol (*p* = 0.206), chewing ability (*p* < 0.001) and speaking ability (*p* = 0.019).

**Table 1:** General characteristics of the study participants.

Continuous variables (*N* = 402)	Mean ± SD	Median (Min-Max)
Age (years)	69.18 ± 5.652	68.00 (61–94)
Categorical variables (*N* = 402)	**Number**	**%**
Age (years)		
61–74	334	83.1
≥ 75	68	16.9
Sex		
Female	247	61.4
Male	155	38.6
Educational level		
>Primary education	338	84.1
≤ Primary education	64	15.9
Monthly income		
≥ 15,000 Thai Baht (USD 475)	230	57.2
<15,000 Thai Baht	172	42.8
Toothbrushing frequency (*N* = 397)		
≥ twice daily	375	94.5
<twice daily	22	5.5
Toothbrushing duration (*N* = 397)		
≥ 2 min	320	80.6
<2 min	77	19.4
Compliance with dental appointments		
Yes	299	74.4
No	103	25.6
Smoking		
Never	393	97.8
Sometimes / Daily	9	2.2
Drinking alcohol		
Never	362	90.0
Occasionally / Daily	40	10.0
Chewing ability		
Comfortable	239	59.5
Fair	142	35.3
Uncomfortable	21	5.2
Speaking ability		
Comfortable	363	90.3
Fair	37	9.2
Uncomfortable	2	0.5
Self-rated oral health		
Good	129	32.1
Fair	222	55.2
Poor	51	12.7

**Notes.**

N number SD standard deviation Min minimum Max maximum USD United States Dollar

**Table 2:** Factors associated with self-rated oral health of the study participants.

**Variables** **(*N* = 402)**	**Self-perceived oral health**		*p* **value**
	**Good/Fair** **N (%)**	**Poor***N* (%)	
Age (years)			0.553
61–74	293 (87.7)	41 (12.3)	
≥ 75	58 (85.3)	10 (14.7)	
Sex			0.168
Female	211 (85.4)	36 (14.6)	
Male	140 (90.3)	15 (9.7)	
Educational level			0.226
>Primary education	298 (88.2)	40 (11.8)	
≤ Primary education	53 (82.8)	11 (17.2)	
Monthly income			0.131
≥ 15,000 Thai Baht (437 USD)	206 (89.6)	24 (10.4)	
<15,000 Thai Baht	145 (84.3)	27 (15.7)	
Toothbrushing frequency (*N* = 397)			0.176
≥ twice daily	330 (88.0)	45 (12.0)	
<twice daily	17 (77.3)	5 (22.7)	
Toothbrushing duration (*N* = 397)			0.572
≥ 2 min	281 (87.8)	39 (12.2)	
<2 min	66 (85.7)	11 (14.3)	
Compliance with dental appointments			0.175
Yes	265 (88.6)	34 (11.4)	
No	86 (83.5)	17 (16.5)	
Smoking			0.319
Never	344 (87.5)	49 (12.5)	
Sometimes/Daily	7 (77.8)	2 (22.2)	
Drinking alcohol			0.206
Never	313 (86.5)	49 (13.5)	
Occasionally/Daily	38 (95.0)	2 (5.0)	
Chewing ability			<0.001^**^
Comfortable (Comfort)	230 (96.2)	9 (3.8)	
Fair/Uncomfortable (Discomfort)	121 (74.2)	42 (25.8)	
Speaking ability			0.019^*^
Comfortable (Comfort)	322 (88.7)	41 (11.3)	
Fair/Uncomfortable (Discomfort)	29 (74.4)	10 (25.6)	

**Notes.**

N number USD United States Dollar

* Fisher’s Exact Test *p* < 0.05.

** Fisher’s Exact Test *p* < 0.01.

Table 3 presents the results of the binary regression analysis. The unadjusted model showed statistically significant associations between poor self-rated oral health and subjective chewing discomfort (*p* < 0.001) along with speaking discomfort (*p* = 0.013). Consequently, after controlling for the other variables in the multivariate model, a significant association remained between poor self-rated oral health and chewing discomfort (*p* < 0.001). The Hosmer-Lemeshow goodness of fit of the final model presented an acceptable *p*-value of 0.930. All VIF estimates were below 1.18, and collinearity tolerance values were greater than 0.847, which did not indicate a multicollinearity problem.

**Table 3:** Simple bivariate and multivariate binary logistic regression analyses of self-rated oral health of the study participants.

**Variables** (*N* = 402)	**Crude OR** **(95% CI)**	***p*** **value**	**Adjusted OR** **(95% CI)**	***p*** **value**
Sex		0.154		0.372
Female	1		1	
Male	0.628 (0.331–1.190)		0.719 (0.348–1.484)	
Educational level		0.241		0.835
>Primary education	1		1	
≤ Primary education	1.546 (0.746–3.204)		1.094 (0.472–2.535)	
Monthly income		0.119		0.180
≥ 15,000 Thai Baht	1		1	
<15,000 Thai Baht	1.598 (0.886–2.882)		1.602 (0.805–3.187)	
Toothbrushing frequency (*N* = 397)		0.149		0.525
≥ twice daily	1		1	
<twice daily	2.157 (0.759–6.131)		1.450 (0.461–4.564)	
Compliance with dental appointments		0.179		0.672
Yes	1		1	
No	1.541 (0.820–2.896)		1.165 (0.575–2.357)	
Drinking alcohol		0.142		0.359
Never	1		1	
Occasionally/Daily	0.336 (0.079–1.438)		0.482 (0.101–2.290)	
Chewing ability		<0.001^**^		<0.001^**^
Comfort	1		1	
Discomfort	8.871 (4.178–18.83)		8.139 (3.756–17.64)	
Speaking ability		0.013^*^		0.309
Comfort	1		1	
Discomfort	2.708 (1.231–5.960)		1.588 (0.652–3.868)	

**Notes.**

N number OR (95% CI) Odd Ratio (95% Confident Interval); 15,000 Thai Baht (437 United States Dollar)

* *p* < 0.05.

** *p* < 0.01.

## Discussion

The present study highlights a strong relationship between self-rated oral health and subjective evaluation of oral functions among elderly patients who attended a dental visit at the university dental hospital in 2020. The final model revealed that chewing discomfort was related to worse self-rated oral health among study participants. There are very few studies among elderly patients in a tertiary care setting that used a global self-rating of oral health assessment. Therefore, we primarily sought to compare most of our findings with other single-item self-rated oral health studies among older participants in various contexts.

Our study found significant associations between self-rated oral health and subjective oral functions (chewing ability and speaking ability). Simple bivariate analyses showed that participants with discomfort chewing were 8.871 times more likely to self-report poor oral health than those who did not report discomfort chewing. Similarly, those who reported discomfort speaking were 2.708 times more likely to self-report poor oral health compared to those who did not report discomfort speaking. These findings are consistent with a prior survey study by Moon, Heo & Jung (2020), which reported that older Koreans with poor subjective masticatory function or pronounciation difficulties were more likely to self-reporting poor oral health.

However, our multivariate analysis showed that only chewing ability was significantly associated with self-rated oral health among older Thai adults. In our finding, participants who reported chewing discomfort were 8.139 times more likely to report poor self-rated oral health than participants who felt comfortable chewing. A possible explanation for this finding may be sampling bias: all participants in the present study were patients who attended at least one dental visit in the past year, so may have been more likely to have oral health problems for which they sought treatment in the past year. Previous studies have found that older adults with dental problems, such as cavities, toothaches, or broken fillings, who may feel uncomfortable eating or having chewing difficulties. As a result, they were more likely to perceive the need for dental care and self-report poor oral health (Ugarte Cabo et al., 2006; Ugarte et al., 2007).

Our study found that most elderly patients attending dental visits at the university dental hospital used subjective oral functions, mainly chewing ability, as a reference point for their self-rated oral health. Recent studies have found that perceived discomfort or pain when chewing can induce poor general health and lower well-being among the elderly. For example, a study in the Netherlands by Kiesswetter et al. (2019) concluded that chewing difficulty was a determinant of nutritional deficiency among community-dwelling older adults. In Finland, a study by Lindroos et al. (2019) found that oral health difficulties, such as chewing discomfort, were associated with psychological well-being, gastrointestinal symptoms, and malnutrition.

Chewing discomfort can even lead to increased mortality among institutionalized older residents (Lindroos et al., 2019) or among older adults living in a community (Okura, Ogita & Arai, 2020). Furthermore, a prior study in Brazil by Saintrain et al. (2018) identified that chewing difficulty as a potential risk factor for dependence on activities of daily living among older adults attending public primary health care centers. Reported discomfort when eating (Ramsay et al., 2018) and self-rated poor oral health (Hakeem, Bernabé & Sabbah, 2021) may also be able to predict frailty risk in older adults.

Our study adds to the evidence that a single-item self-measurement can predict oral and general health risk in tertiary care institutions, as well as primary care settings and community-based survey research. It can also be used as a standard measure for preliminary screening in conjunction with the patient’s chief complaints.

Our study did not show an association between self-rated oral health and socioeconomic status or oral health-related behaviors. Our findings are also consistent with several studies that did not report an association between self-reported oral health and age or sex (Dahl, Calogiuri & Jönsson, 2018; Ekanayke & Perera, 2005; Ugarte Cabo et al., 2006; Ugarte et al., 2007). Because we collected data among elderly patients from a single university dental hospital in the capital of Thailand, our study participants were more likely to share certain demographic characteristics such as socioeconomic status. Our participants also had similar oral health-related behaviors, as most of them were long-time patients of the same university dental hospital.

For self-perceived oral health, 32.1% of the participants in our study reported “good” self-rated oral health, 40.5% reported chewing discomfort, and 9.7% reported speaking discomfort. In comparison, in the 8th NSOHT, a national survey conducted among older Thai adults living in the community, just 24.9% of respondents reported “good” self-rated oral health, and more people reported problems chewing (52.6%) and speaking (12.6%) than in our study (MPH et al., 2018). These findings confirm that the elderly in the two surveys who had chewing and speaking problems were more likely to report poor oral health. However, comparable variables revealed that the participants in our study had larger incomes, a higher level of education, a longer toothbrushing duration, and more regular dental visits than those who participated in the national survey ( MPH et al., 2018). Therefore, our participants had a higher frequency of positive self-rated oral health.

Our observations are in line with the literature that indicates that people who resided in rural regions are less likely to report good oral health, and those who lived in metropolitan areas with higher incomes are more likely to self-rate positive oral health (Martins et al., 2011; Ugarte et al., 2007). Lower economic status and limited education are also associated with poor self-reported oral health and uncomfortable oral functions in older adults (Aida et al., 2011; Ekanayke & Perera, 2005; Ugarte et al., 2007). Additionally, a lower level of education also induces negative oral health behaviors in the elderly (Moon, Heo & Jung, 2020; Ugarte et al., 2007).

In general, preventive dentistry and oral health promotion programs improve oral health-related behaviors. In addition, oral health education interventions help improve self-care ability in oral health and reduce barriers to using oral health services among older people (Petersen & Yamamoto, 2005). Therefore, good oral health promotion programs are needed in the older adult population.

### Limitations

Some limitations deserve to be mentioned. The present study uses a cross-sectional research design, so a causal relationship cannot be drawn. We also focused on the university dental hospital as a single health unit, so most of the participants could have similar sociodemographic characteristics and oral health-related behaviors. The participants self-reported their information, and we did not perform an oral examination, which was beyond the scope of this study.

We also reached our needed sample size with a 48.1% call response rate. A total of 31.1% of the incomplete calls were because there was no answer, it continued to ring, the call went to voicemail, or the telephone number was invalid. We found that women were more willing to participate in and complete the phone interview than men, so 61.4% of our participants were older women. We also found that those over 75 years of age, especially men, felt less comfortable participating in a telephone interview, so 83.1% of our participants were under 75 years old. Therefore, our sample is not representative of all patients in the university dental hospital or the entire older adult population in Thailand. Moreover, our study focused only on two oral functions—chewing and speaking—and did not involve physical conditions, the effects of modifications because of those conditions, emotions, health behaviors, or other background factors. Survey responses could also simply reflect the quality of dental services in the university dental hospital. These limitations can impede the generalization of the findings and should be addressed in future studies.

## Conclusions

Despite the limitations, the findings of this study are essential because the results have indicated that elderly patients with chewing discomfort were less likely to self-report good oral health. Difficulty chewing could be a potential factor influencing poor self-rated oral health among older patients attending the university dental hospital. Therefore, healthcare providers should routinely evaluate self-rated oral health among elderly patients to detect early signs and symptoms of oral health problems, assess the success of dental treatments, and monitor general health and well-being. Additionally, it is necessary to develop educational oral health promotion programs to improve the quality of routine oral self-care, which can help older adults retain an adequate number of teeth for comfortable oral functions and achieve good oral health.

##  Supplemental Information

10.7717/peerj.14191/supp-1 Supplemental Information 1 The raw measurements The raw data were used for statistical analysis to explore the potential factors associated with self-rated oral health among elderly patients attending a university dental hospital in Thailand.

10.7717/peerj.14191/supp-2 Supplemental Information 2 SPSS codebook

## Additional Information and Declarations

### Data Availability

The following information was supplied regarding data availability: The raw data is available in the Supplementary Files.
