# Supplementary material for: Self-rated oral health among elderly patients attending a university dental hospital in Thailand: a telephone-based cross-sectional survey study
Source: PeerJ. 2022 Oct 10;10:e14191. doi: 10.7717/peerj.14191 (PMC9558616; doi:10.7717/peerj.14191)
Supplement: Supplemental Information 2 [file peerj-10-14191-s002.pdf]

File Information

Notes

Output Created		13-MAY-2022 10:16:28
Comments		
Input	Data	C:\Users\Nithimar\Desktop\SPSS Research.sav
	Active Dataset	DataSet1
	Filter	<none>
	Weight	<none>
	Split File	<none>
	N of Rows in Working Data File	402
Syntax		DISPLAY DICTIONARY.
Resources	Processor Time	00:00:00.00
	Elapsed Time	00:00:00.00

Variable Information

Variable	Position	Label	Measurement Level	Role	Column Width	Alignment	Print Format	Write Format	Missing Values
ID	1	ID	Scale	Input	8	Right	F8	F8	
Age	2	Age	Scale	Input	8	Right	F8	F8	
AgeGroup	3	Age group	Nominal	Input	8	Right	F8	F8	
Sex	4	Sex	Nominal	Input	8	Right	F8	F8	
Education	5	Educational level	Nominal	Input	8	Right	F8	F8	
Income	6	Monthly income	Nominal	Input	8	Right	F8	F8	
BrushFrequency	7	Brushing frequency	Nominal	Input	8	Right	F8	F8	9
BrushDuration	8	Brushing duration	Nominal	Input	8	Right	F8	F8	9
DentalCare	9	Dental Care	Nominal	Input	8	Right	F8	F8	
Smoking	10	Smoking	Nominal	Input	8	Right	F8	F8	
Drinking	11	Drinking	Nominal	Input	8	Right	F8	F8	
Chewing	12	Chewing ability	Ordinal	Input	8	Right	F8	F8	
ChewingGroup	13	Chewing group	Nominal	Input	8	Right	F8	F8	
Speaking	14	Speaking ability	Ordinal	Input	8	Right	F8	F8	
SpeakingGroup	15	Speaking group	Nominal	Input	8	Right	F8	F8	
SROH	16	Self-rated oral health	Ordinal	Input	8	Right	F8	F8	
SROH_gr	17	SROH group	Nominal	Input	8	Right	F8	F8	

Variables in the working file

Variable Values

Value		Label
AgeGroup	0	64-74
	1	>=75
Sex	0	Female
	1	Male
Education	0	>Primary education
	1	<= Primary education
Income	0	>=15000 Baht (USD 475)
	1	< 15000 Baht
BrushFrequency	0	>= twice daily
	1	< twice daily
BrushDuration	0	>= 2 minutes
	1	< 2 minutes
DentalCare	0	Yes
	1	No
Smoking	0	Never
	1	Sometimes/Daily
Drinking	0	Never
	1	Occationally/Daily
Chewing	0	Comfortable
	1	Fair
	2	Uncomfortable
ChewingGroup	0	Comfortable
	1	Fair/Uncomfortable
Speaking	0	Comfortable
	1	Fair
	2	Uncomfortable
SpeakingGroup	0	Comfortable
	1	Fair/Uncomfortable
SROH	0	Good
	1	Fair
	2	Poor
SROH_gr	0	Good / Fair
	1	Poor
